# Natural Bis-Benzylisoquinoline Alkaloids-Tetrandrine, Fangchinoline, and Cepharanthine, Inhibit Human Coronavirus OC43 Infection of MRC-5 Human Lung Cells

**DOI:** 10.3390/biom9110696

**Published:** 2019-11-04

**Authors:** Dong Eon Kim, Jung Sun Min, Min Seong Jang, Jun Young Lee, Young Sup Shin, Chul Min Park, Jong Hwan Song, Hyoung Rae Kim, Seungtaek Kim, Young-Hee Jin, Sunoh Kwon

**Affiliations:** 1 Herbal Medicine Research Division, Korea Institute of Oriental Medicine, Daejeon 34054, Korea; ehddjs0@kiom.re.kr (D.E.K.); jsmin1019@kiom.re.kr (J.S.M.); 2 Center for Convergent Research of Emerging Virus Infection, Korea Research Institute of Chemical Technology, Daejeon 34114, Korea; minseongjang@kitox.re.kr (M.S.J.); ljy3695@krict.re.kr (J.Y.L.); pong1140@krict.re.kr (Y.S.S.); parkcm@krict.re.kr (C.M.P.); jhsong@krict.re.kr (J.H.S.); hyungrk@krict.re.kr (H.R.K.); 3 Department of Non-Clinical Studies, Korea Institute of Toxicology, Daejeon 34114, Korea; 4 Zoonotic Virus Laboratory, Institute Pasteur Korea, Seongnam 13488, Korea; seungtaek.kim@ip-korea.org; 5 KM Application Center, Korea Institute of Oriental Medicine, Daegu 41062, Korea

**Keywords:** bis-benzylisoquinoline alkaloids, tetrandrine, fangchinoline, cepharanthine, human coronavirus strain OC43, MRC-5 human lung cell, antiviral effect

## Abstract

*Stephania tetrandra* and other related species of *Menispermaceae* are the major sources of the bis-benzylisoquinoline alkaloids tetrandrine (TET), fangchinoline (FAN), and cepharanthine (CEP). Although the pharmacological properties of these compounds include anticancer and anti-inflammatory activities, the antiviral effects of these compounds against human coronavirus (HCoV) remain unclear. Hence, the aims of the current study were to assess the antiviral activities of TET, FAN, and CEP and to elucidate the underlying mechanisms in HCoV-OC43-infected MRC-5 human lung cells. These compounds significantly inhibited virus-induced cell death at the early stage of virus infection. TET, FAN, and CEP treatment dramatically suppressed the replication of HCoV-OC43 as well as inhibited viral S and N protein expression. The virus-induced host response was reduced by compound treatment as compared with the vehicle control. Taken together, these findings demonstrate that TET, FAN, and CEP are potential natural antiviral agents for the prevention and treatment of HCoV-OC43 infection.

## 1. Introduction

Natural products are regarded as good sources for the development of antiviral agents [1]. Various medicinal herbs with anti-inflammatory, antifungal, and antitumor activities have been widely studied to identify those with antiviral properties [2,3,4]. *Stephania tetrandra* and other related species of *Menispermaceae* are the major sources of the bis-benzylisoquinoline alkaloids tetrandrine (TET), fangchinoline (FAN), and cepharanthine (CEP). These herbal plants have been traditionally used for various medicinal purposes in the East Asian countries [5]. Weber and Opatz demonstrated that the bioreactive properties of these bis-benzylisoquinoline alkaloids include anticancer, anti-inflammatory, and anti-oxidative activities [6]. TET exhibits broad pharmacological actions that include anti-inflammatory effects as well as immunosuppressant and anticancer activities [5]. Several studies have reported the effects of TET against the infection of different types of viruses such as herpes simplex virus, dengue virus, and Ebola virus [7,8,9]; others have shown that FAN inhibits the replication of human immunodeficiency virus type 1 (HIV-1) [10] and that CEP possesses antiviral activities against HIV-1 [11] and herpes simplex virus type 1 [12]. Coronaviruses (CoVs) are enveloped, positive-sense, single-stranded RNA viruses that infect a broad range of animal species and cause multiple respiratory outcomes of varying severity, including the common cold, bronchiolitis, and pneumonia [13]. CoVs are subdivided into four genera (Alpha-, Beta-, Gamma-, and Delta-) [14]. Among the six CoVs isolated from humans [15], the World Health Organization declared that accelerated research and the development of antivirals for the treatment of emerging zoonotic viruses, including β-CoVs, Middle East respiratory syndrome-related coronavirus (MERS-CoV), and severe acute respiratory syndrome-related coronavirus (SARS-CoV), are urgently needed [16]. Since the mid-1960s, human coronavirus strains OC43 (HCoV-OC43; β-CoV) and 229E (α-CoV) have been considered as mostly responsible for the common cold [17,18]. Notably, HCoV-OC43, which is the most prevalent subtype of HCoV [19], is responsible for up to 30% of respiratory infections and can cause repeated reinfections throughout life [20,21]. Moreover, HCoV-OC43 is most closely related to SARS-CoV and MERS-CoV, and shares several functional properties with both [22,23]. Due to the similarities with SARS-CoV and MERS-CoV, HCoV-OC43 has been used as an alternative model for research of these emerging viral strains to avoid the limitation of the requirement for a biosafety level 3 (BSL-3) facility.

The aim of the present study was to investigate the antiviral activities of TET, as a herb-derived, small-molecule compound, in HCoV-OC43-infected MRC-5 fibroblasts derived from human lung tissue. The results showed that TET inhibited HCoV-OC43 infection of MRC-5 cells in a dose-dependent manner. In addition, the antiviral effects of FAN and CEP, which are also natural compounds with chemical structures similar to that of TET, were verified. Overall, our findings suggest that TET, FAN, and CEP are potential antiviral candidates for the treatment of human β-CoV infection.

## 2. Materials and Methods 

### 2.1. Preparation of Compounds

TET (PubChem CID: 73078), FAN (PubChem CID: 73481), and CEP (PubChem CID: 10206) were purchased from Wuhan ChemFaces Biochemical Co., Ltd. (Wuhan, China), dissolved in dimethyl sulfoxide (DMSO), and stored as 20 mM stock solutions at −80 °C. Each compound was freshly prepared to the indicated concentrations with fetal bovine serum (FBS)-free culture medium before use. The concentration of dimethyl sulfoxide in this experiment did not exceed 0.05%.

### 2.2. Human Cell Line Culture

MRC-5 cells (American Type Culture Collection (ATCC), Manassas, VA, USA) were grown in minimal essential medium (MEM; Corning Incorporated, Corning, NY, USA) supplemented with 10% heat-inactivated FBS (WelGENE, Gyeongsan-si, South Korea), 100 U/mL of penicillin, and 100 μg/mL of streptomycin (Gibco, Carlsbad, CA, USA). The cells were seeded in the wells of 96-well plates (Thermo Fisher Scientific, Waltham, MA, USA) at 1.5 × 10^4^ cells/well or 24-well plates (Corning Incorporated) at 8 × 10^4^ cells/well and cultured at 37 °C under a humidified atmosphere of 5% CO_2_.

### 2.3. HCoV-OC43 Propagation

HCoV-OC43 (ATCC; 10^10^ copies/μL) was propagated in MRC-5 cells that reached 90% confluency in MEM supplemented with 2% heat-inactivated FBS, 100 U/mL of penicillin, and 100 μg/μL of streptomycin at 33 °C under a humidified atmosphere of 5% CO_2_. When the cells reached 50% confluence, the supernatant was harvested and filtered with a 0.22-μm Stericup^®^ Filter Unit (EMD Millipore Corporation, Billerica, MA, USA). Aliquots of filtered supernatants were frozen at −80 °C until required.

### 2.4. Virus Titration

Confluent MRC-5 cells (1.5 × 10^4^) were cultured in the wells of 96-well plates (100 μL/well). Serial 10-fold dilutions of the HCoV-OC43 stocks (10^−1^ to 10^−8^) were prepared in MEM supplemented with 2% heat-inactivated FBS, 100 U/mL of penicillin, and 100 μg/mL of streptomycin at 33 °C under a humidified atmosphere of 5% CO2 for four days. At four days post-infection (dpi), the cytopathic effect of the infected cells was quantified by crystal violet staining. The titer of the purified HCoV-OC43 was 10^6.5^ TCID_50_ (median tissue culture infectious dose)/100 μL.

### 2.5. Determination of the Half Maximal Inhibitory Concentration (IC_50_) of TET, FAN, and CEP

MRC-5 cells (1 × 10^4^) were seeded in the wells of 96-well tissue culture plates. The next day, the cells were infected with 10^3.5^ TCID_50_ HCoV-OC43 and treated with two-fold serial dilutions of the compounds (from 10 μM). At four dpi, the cell viability of each group was determined using the MTS [3-(4,5-dimethylthiazol-2-yl)-5-(3-carboxymethoxyphenyl)-2-(4-sulfophenyl)-2*H*-tetrazolium] assay. The IC_50_ value was determined by nonlinear regression analysis.

### 2.6. Time-of-Addition Assay

MRC-5 cells (1 × 10^4^) were seeded in the wells of 96-well tissue culture plates. The next day, the cells were pretreated with the compounds at concentrations of 0.2, 1, and 5 μM. After 24 h, the compounds were removed by washing, and the cells were infected with HCoV-OC43 for four days. Then, the compounds were added to the MRC-5 cell cultures either during (co-treatment) or 24 h after infection (post-treatment). As an additional experiment, the compounds (5 μM) were added to the MRC-5 cell cultures at 24, 12, 6, and 3 h before infection (pretreatment). The MRC-5 cells were then treated with the compounds either during infection (co-treatment) or 12, 24, and 48 h post-infection (post-treatment). At four dpi, the cell viability of each group was determined using the MTS assay.

### 2.7. MTS Assay of Cell Viability

Cytotoxicity was measured using the CellTiter 96^®^ AQueous One Solution Cell Proliferation Assay (Promega Corporation, Madison, WI, USA) in accordance with the manufacturer’s protocol. Briefly, the medium was harvested, and 90 μL of MEM supplemented with 2% heat-inactivated FBS, 100 U/mL of penicillin, 100 μg/mL of streptomycin, and 10 μL of MTS reagent (Promega Corporation, Madison, WI, USA) were added to each well. After incubation for 2 h at 37 °C under a humidified atmosphere of 5% CO_2_, the optical density of each well was measured at a wavelength of 490 nm using a GloMax^®^ Discover Microplate Reader (Promega Corporation).

### 2.8. Quantification of the Copy Number of HCoV-OC43 RNA

The sequence of the HCoV-OC43 nucleoprotein (N protein; 138 bp) coded by the pBIC-A vector (DNA concentration, 1 μg) was linearized with the *Xho*I restriction enzyme and purified with the HiYield Plus™ Plasmid Mini Kit (Real Biotech Corporation, Banqiao City, Taipei County, Taiwan). Subsequently, RNA transcripts were obtained using the Invitrogen™ MEGAscript™ T7 Transcription Kit (Thermo Fisher Scientific, Waltham, MA, USA) according to the manufacturer’s protocol for standards. Purification of viral RNA in the filtered culture supernatants was performed using a commercial viral RNA purification kit (Qiagen, Hilden, Germany) according to the manufacturer’s instructions. Ten-fold serial dilutions of the N protein RNA (10^10^–10^1^ copies/μL) as standards and purified HCoV-OC43 RNA were synthesized to complementary DNA. Quantitative reverse transcription PCR (qRT-PCR) was performed using the One Step SYBR^®^ PrimeScriptTM RT-PCR Kit (Takara Bio, Inc., Shiga, Japan) according to the manufacturer’s protocol by means of the following primer pair for the HCoV-OC43 N gene: (forward) 5′-AGCAACCAGGCTGATGTCAATACC and (reverse) 5′-AGCAGACCTTCCTGAGCCTTCAAT. The amplification condition was set at 40 cycles at 95 °C for 5 s and at 60 °C for 30 s using a LightCycler^®^ 96 Instrument (Roche Life Science, Penzberg, Germany). The copy number was calculated using a standard curve of HCoV-OC43 RNA.

### 2.9. Cytokine mRNA Quantification by qRT-PCR

MRC-5 cells (1 × 10^5^) were seeded in the wells of 24-well tissue culture plates. The next day, the cells were infected with HCoV-OC43, and each compound (5 μM) was added to the wells. Total RNA was isolated from MRC-5 cells at the designated time using the RNeasy^®^ Mini kit (Qiagen, Hilden, Germany) according to the manufacturer’s instructions. Total RNA was synthesized to complementary DNA using the One Step SYBR® PrimeScriptTM RT-PCR Kit (Takara Bio, Inc., Shiga, Japan) according to the manufacturer’s instructions. The sequences of the human-specific primers used for qRT-PCR are listed in Table 1.

### 2.10. Western Blot Analysis

After washing once with Dulbecco’s phosphate-buffered saline (PBS), the cells were lysed in Glo lysis buffer (Promega Corporation) for 5 min at 4 °C. The protein concentration in total cell lysate was measured using a Bio-Rad protein assay kit (Bio-Rad Laboratories, Hercules, CA, USA). Then, a 20-μg aliquot of the extracted protein was separated by 8% sodium dodecyl sulfate-polyacrylamide gel electrophoresis and transferred to nitrocellulose membranes (Bio-Rad Laboratories, Hercules, CA, USA) using a Trans-Blot^®^ SD Semi-Dry Electrophoretic Transfer Cell (Bio-Rad Laboratories, Hercules, CA, USA). The membranes were blocked by incubation with 5% skim milk for 1 h at room temperature (RT). After washing three times with Tris-buffered saline with Tween 20 (TBST), the membranes were incubated with primary antibodies against the HCoV-OC43 spike glycoprotein (Cusabio Technology LLC, Houston, TX, USA) and α-tubulin (Cell Signaling Technology, Inc., Danvers, MA, USA) at 4 °C overnight. After washing three times with TBST, the membranes were incubated with horseradish peroxidase-conjugated secondary antibodies for 1 h at RT. Afterward, signals were detected with Pierce™ Enhanced Chemiluminescence Western Blotting Substrate (Thermo Fisher Scientific, Waltham, MA, USA) using the ChemiDoc™ Touch Imaging System (Bio-Rad Laboratories, Hercules, CA, USA).

### 2.11. Cytometric Bead Array (CBA) of Cytokines

The detection of six inflammatory cytokines [i.e., interleukin (IL)-1β, IL-6, IL-8, IL-10, IL-12p70, and tumor necrosis factor (TNF)] in the culture supernatants of MRC-5 cells was performed using a BD™ CBA Human Inflammatory Cytokines Kit (BD Biosciences, San Jose, CA, USA). Data were analyzed using FCAP Array™ Software version 3.0 (Soft Flow Hungary, Ltd., Pécs, Hungary) with standard curves of recombinant cytokine standards.

### 2.12. Immunohistochemical Analysis

MRC-5 cells were fixed with 4% paraformaldehyde and washed with ice-cold PBS. The fixed cells were permeabilized with PBS containing 0.2% Triton X-100 (Sigma-Aldrich Corporation, St. Louis, MO, USA) for 10 min at RT. After blocking with PBS containing 3% bovine serum albumin and 0.2% triton X 100 for 30 min at RT, the cells were incubated with a monoclonal primary antibody against the HCoV-OC43 N protein (dilution, 1:500; EMD Millipore Corporation) at 4 °C overnight. After rinsing with PBS, the cells were incubated with Alexa Fluor^®^ 555 goat anti-mouse secondary antibody (dilution, 1:1000; Invitrogen Corporation, Carlsbad, CA, USA) for 1 h at RT. The stained cells were mounted on slides with SlowFade^®^ Gold Antifade Mountant with 4′,6-diamidino-2-phenylindole (DAPI; Invitrogen Corporation, Waltham, MA, USA). Images were captured using a fluorescence microscope (Olympus IX71; Olympus Corporation, Tokyo, Japan) and analyzed using MetaMorph Image Analysis Software (Molecular Devices, Sunnyvale, CA, USA).

### 2.13. Statistical Analysis

Statistical analysis was performed using GraphPad Prism Software V.6.05 for Windows (GraphPad Software, Inc., San Diego, CA, USA). Data are presented as mean ± standard error of the mean (SEM). Probability (p) values were calculated by one-way analysis of variance (ANOVA) followed by Tukey’s test as indicated in the figure legends (* *p* < 0.05, ** *p* < 0.01, *** *p* < 0.001, and **** *p* < 0.0001).

## 3. Results

### 3.1. TET, FAN, and CEP Protected MRC-5 Cells from the Cytopathic Effect of HCoV-OC43

**Figure 1:**
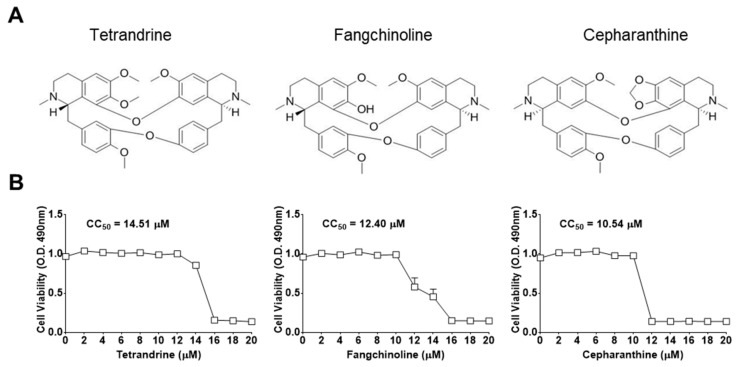
Chemical structures and the 50% cytotoxic concentration (CC_50_) values of bis-benzylisoquinoline alkaloids tetrandrine (TET), fangchinoline (FAN), and cepharanthine (CEP). (**A**) Chemical structures of TET, FAN, and CEP. (**B**) MRC-5 cells were cultured from 20 μM for four days. Cell viability was assessed using the 3-(4,5-dimethylthiazol-2-yl)-5-(3-carboxymethoxyphenyl)-2-(4-sulfophenyl)-2H-tetrazolium (MTS) assay, and the cytotoxic concentration (CC_50_) values of the compounds were calculated. Data are presented as mean ± SEM of three independent experiments.

**Figure 2:**
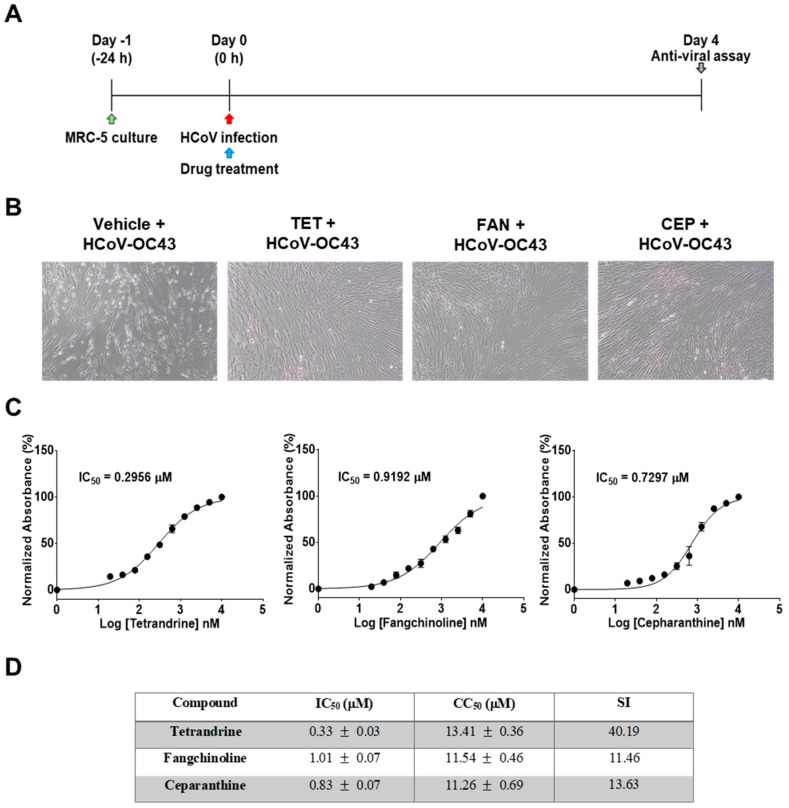
Antiviral activities of TET, FAN, and CEP in MRC-5 cells. MRC-5 cells were infected with human coronavirus (HCoV)-OC43 with or without the addition of two-fold serial dilutions of the compounds. (**A**) Overall scheme for MRC-5 cell infection and treatment with TET, FAN, and CEP. (**B**) Live photo image of MRC-5 at four dpi. (**C**) The inhibitory concentration (IC_50_) values of the compounds were calculated at four dpi by nonlinear regression analysis. (**D**) Lists the IC_50_, cytotoxic concentration (CC_50_), and selectivity indices (CC_50_/IC_50_, SI) values. Data are presented as mean ± SEM of three independent experiments.

First, the cytotoxic effects of TET, FAN, and CEP in MRC-5 cells were determined using the MTS assay. The 50% cytotoxic concentration (CC_50_) values of the compounds were 14.51, 12.40, and 10.54 μM, respectively (Figure 1). Then, the antiviral effects of TET, FAN, and CEP were examined. Briefly, MRC-5 cells were cultured with two-fold serial dilutions of each compound (from 10 μM) and then infected with HCoV-OC43. The cytopathic effect of HCoV-OC43 in MRC-5 cells was determined at four dpi (Figure 2A). Live photo imaging showed HCoV-OC43-induced cell death in the vehicle-treated group (Figure 2B). In contrast, the MRC-5 cells in the TET, FAN, and CEP treatment groups remained morphologically unchanged. As shown in Figure 2C, compound treatment dose-dependently increased cell viability against HCoV-OC43 infection. In addition, no cytotoxicity was caused by the compounds within the effective ranges, even at a concentration as high as 10 μM (CC_50_ > 10 μM). The IC_50_ values of TET, FAN, and CEP were 295.6, 919.2, and 729.7 nM, respectively, which resulted in selectivity indices (CC_50_/IC_50_) of > 40, 11, and 13, respectively (Figure 2D), suggesting that the antiviral activity of TET was more effective than that of FAN and CEP. Together, these data show that treatment with an effective dose of TET, FAN, and CEP has potential anti-HCoV-OC43 activities.

### 3.2. TEN, FAN, and CEP Have Antiviral Activities at the Early Stage of HCoV-OC43 Infection

**Figure 3:**
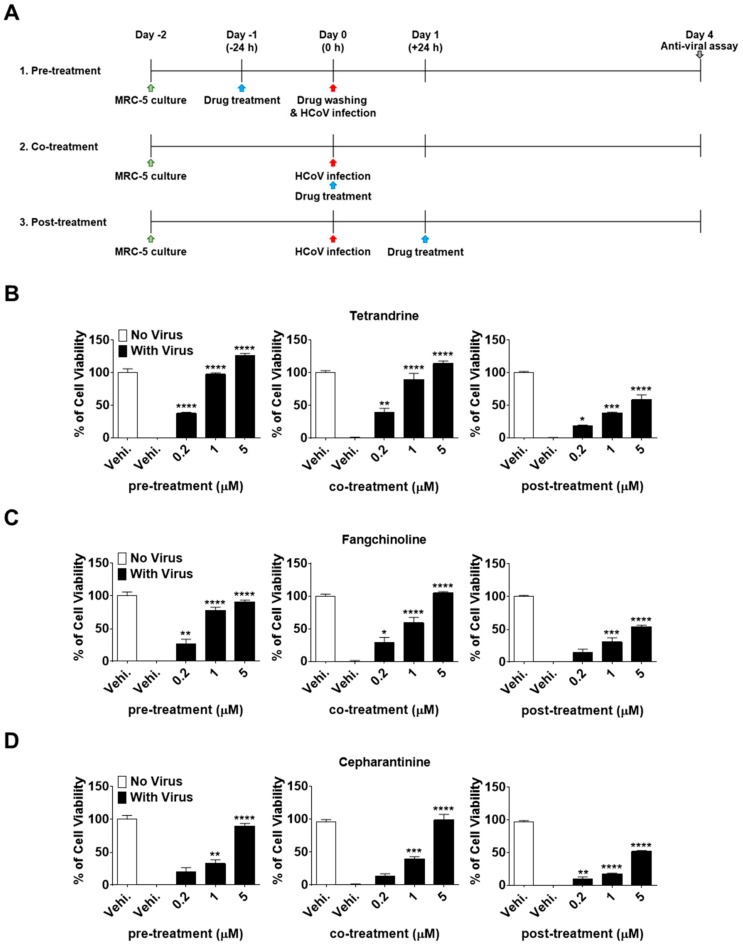
Dose- and time-dependent antiviral activities of TET, FAN, and CEP. MRC-5 cells were infected with HCoV-OC43 with or without the addition of TET, FAN, and CEP at the indicated concentrations (Vehicle 0.025% dimethyl sulfoxide (DMSO)). The compounds were added at 24 h before, during, or 24 h after HCoV-OC43 infection of MRC-5 cells. (**A**) Times of compound addition to infected MRC-5 cells. (**B**–**D**) Cell viability of pre-, co-, post- compound treated cells was assessed using the MTS assay at four dpi. Data are presented as mean ± SEM of three independent experiments. * *p* < 0.05, ** *p* < 0.01, *** *p* < 0.001, and **** *p* < 0.0001 versus the vehicle-treated virus-infected group (one-way ANOVA plus Tukey’s multiple comparisons test).

To investigate the effectiveness of TET, FAN, and CEP at different stages of the HCoV-OC43 life cycle, time-of-addition assays were performed. Briefly, MRC-5 cells were treated with 0.2, 1, and 5 μM of the compounds at 24 h before infection with HCoV-OC43 (pre-treatment). After washing to remove the compounds, MRC-5 cells were infected with HCoV-OC43 and cultured for four days. Additionally, compounds were added to the MRC-5 cell cultures during infection (co-treatment) or 24 h after infection (post-treatment) (Figure 3A). Interestingly, compound administration at 24 h after treatment significantly increased MRC-5 cell survival in a dose-dependent manner at a rate comparable to that of the co-treatment group (Figure 3B–D). However, when administered at 24 h post-treatment, the cell viability did not increase as much as in the pre- and co-treatment groups. Overall, these results demonstrate that TEN, FAN, and CEP have dose- and time-dependent anti-HCoV-OC43 activities. The cell survival against virus infection was more efficiently increased by the pre- and co-exposure of compounds than by exposure after infection.

**Figure 4:**
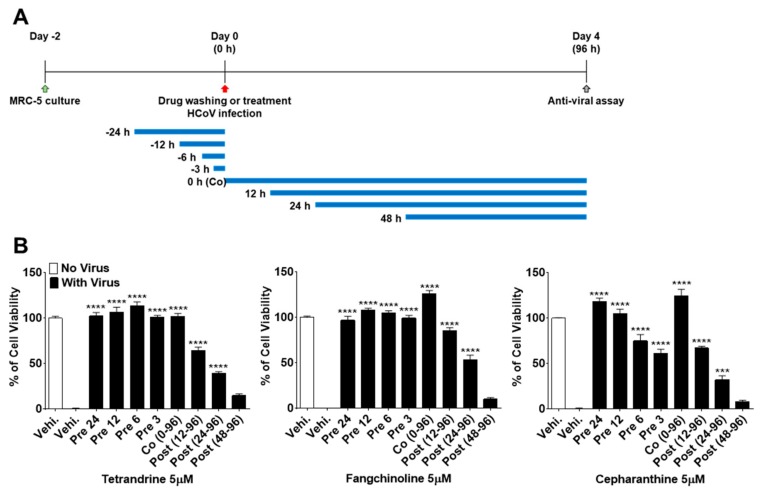
Time course assay of TET, FAN, and CEP. MRC-5 cells were treated with 5 μM compounds (Vehicle 0.025% DMSO) for 24 h (pre 24), 12 h (pre 12), 6 h (pre 6), and 3 h (pre 3) prior to HCoV-OC43 infection or during infection (co 0–96), at 12 h (post 12–96), 24 h (post 24–96), and 48 h (post 48–96) post-infection. (**A**) The overall scheme for time-of-additional assay. The blue lines refer to the compound administration period. (**B**) The cell viability was assessed by MTS-based assay at four dpi. Data are presented as means ± SEM and are representative of three independent experiments. *** *p* < 0.001, **** *p* < 0.0001 (One-way ANOVA plus Tukey’s multiple comparisons test) compared with vehicle group.

To more precisely verify the antiviral effects of TEN, FAN, and CEP over time, MRC-5 cells were treated with 5 μM concentrations of the compounds at 24, 12, 6, and 3 h before HCoV-OC43 infection, during infection, and at 12, 24, and 48 h post-infection (Figure 4A). As shown in Figure 4B, the viability of MRC-5 cells treated with the compounds before virus infection was increased as compared with that of the control vehicle group. Notably, TET, FAN, and CEP had highly effective antiviral activities for only 3 h of treatment before HCoV-OC43 infection, although CEP (61.1%) was not as effective as TET and FAN. In contrast, the viability of cells treated with the three compounds at 48 h after infection was similar to that of the untreated group, suggesting no antiviral effect at 48 h after infection. Together, these data suggest that TET, FAN, and CEP have antiviral activities for only 3 h of treatment before HCoV-OC43 infection and for 48 h of treatment after infection. These results indicate that TET, FAN, and CEP are most effective at the early stage of the HCoV-OC43 life cycle, and the timing of treatment is more important to protect against virus-induced cell death rather than the duration of cell exposure to the compounds.

### 3.3. TET, FAN, and CEP Inhibited HCoV-OC43 Replication and N Protein Expression in MRC-5 Cells

**Figure 5:**
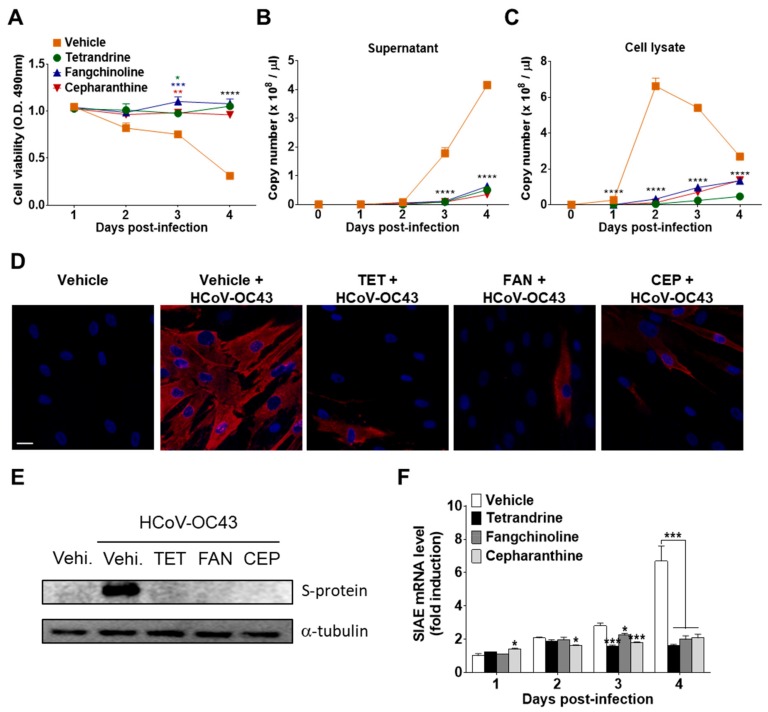
Inhibition of HCoV-OC43 copy number, nucleoprotein (N) and spike glycoprotein (S) protein expression, and sialic acid acetylesterase (SIAE) mRNA levels in MRC-5 cells by TET, FAN, and CEP. HCoV-OC43-infected MRC-5 cells were treated with 5 μM TET, FAN, and CEP (Vehicle 0.025% DMSO). The culture supernatant and cell lysates were harvested at the designated times. (**A**) Cell viability was assessed using the MTS assay at 1, 2, 3, and 4 days post-infection (**B** and **C**). The RNA copy number of the HCoV-OC43 N protein released into the culture supernatant (**B**) and cell lysates (**C**) were examined by qRT-PCR. Viral RNA and total RNA were isolated from culture supernatants and the lysates of infected cells for quantification of HCoV-OC43 RNA levels. (**D**) Images were captured by fluorescence microscopy at two dpi. Immunofluorescence staining of the nucleus with 4′,6-diamidino-2-phenylindole (DAPI) (blue) and HCoV-OC43 N protein (red). Scale bar = 50 μM. (**E**) Immunoblot analysis of the cell lysates at two dpi. The S protein of HCoV-OC43 was detected with α-tubulin as a control. (**F**) SIAE mRNA levels of MRC-5 cells were analyzed at 1, 2, 3, and 4 dpi using qRT-PCR. β-Actin was used for data normalization. Data are presented as mean ± SEM of three independent experiments. * *p* < 0.05, ** *p* < 0.01, *** *p* < 0.001, and **** *p* < 0.0001 versus the vehicle group (one-way ANOVA plus Tukey’s multiple comparisons test).

To investigate the effects of the compounds on virus replication, MRC-5 cells were infected with HCoV-OC43, and the compounds (5 μM) were added (co-treatment). The supernatants and cell lysates were harvested at 1, 2, 3, and 4 dpi. The abundance of viruses in the supernatants and cell lysates was quantified by measuring the HCoV-OC43 N protein RNA levels by qRT-PCR. As shown in Figure 5A,B, virus infection promoted cell death and increased the release of HCoV-OC43 into the supernatants in a time-dependent manner. However, treatment with TET, FAN, and CEP significantly inhibited virus-induced cell death and the release of the virus into the supernatant. Moreover, the copy number of viral RNA peaked at two dpi and then slowly decreased in MRC-5 cell lysates in the vehicle group. However, compound treatment significantly decreased the copy number of viral RNA in cell lysates (Figure 5C). These results show that TET, FAN, and CEP can inhibit the replication of HCoV-OC43 in MRC-5 cells. Next, HCoV-OC43 N protein in the MRC-5 cells was visualized using fluorescence microscopy. As shown in Figure 5D, most of the vehicle-treated cells expressed the N protein at two dpi, whereas treatment with TET, FAN, and CEP highly decreased the expression of the N protein in MRC-5 cells. Additionally, the expression level of the S protein was determined in the lysate of virus-infected MRC-5 cells by western blot analysis (Figure 5E). The expression levels of HCoV-OC43 S protein remarkably increased at two dpi, although the levels were undetectable in the lysate of MRC-5 cells at one dpi (data not shown). However, the S protein was undetectable following compound treatment. In addition, the mRNA levels of sialic acid acetylesterase (SIAE) [24] were dramatically induced by HCoV-OC43 infection in MRC-5 cells at four dpi. The mRNA levels of SIAE following treatment with TET, FAN, and CEP were lower than with the vehicle control (Figure 5F). Collectively, these data suggest that TET, FAN, and CEP inhibited the replication of HCoV-OC43, the expression levels of the N and S proteins, as well as the virus-induced host response, as indicated by SIAE induction in MRC-5 cells.

### 3.4. Antiviral Gene Expression and Inflammatory Cytokine Production Induced by HCoV-OC43 Infection were Reduced in Compound-Treated MRC-5 Cells

**Figure 6:**
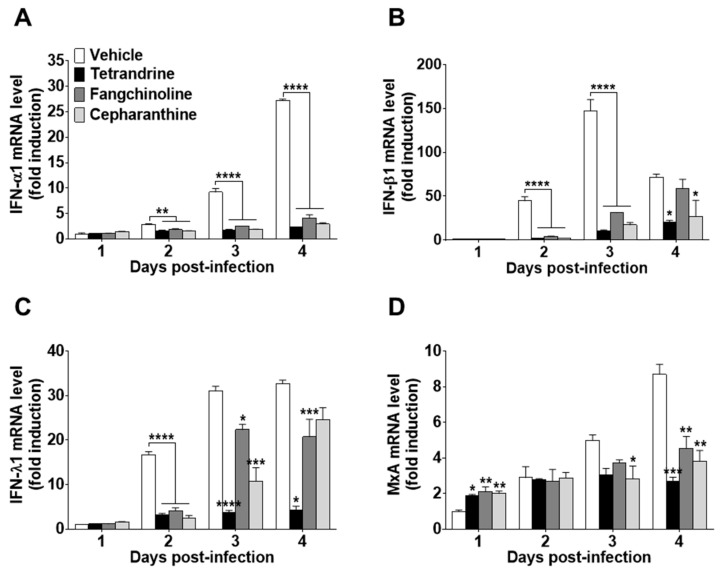
Reduced interferon (IFN)-related gene expression in TET-, FAN-, and CEP-treated HCoV-infected MRC-5 cells. The compounds (5 μM) were added to HCoV-OC43-infected MRC-5 cells. Total RNA was purified from MRC-5 cells at days 1, 2, 3, and 4. (**A–D**) The mRNA levels of (**A**) IFN-α1, (**B**) IFN-β1, (**C**) IFN-λ1, and (**D**) MxA genes were measured by qRT-PCR in vehicle (0.025% DMSO), TET-, FAN-, and CEP-treated cell lysates. β-Actin was used for data normalization. Data are presented as mean ± SEM of three independent experiments. * *p* < 0.05, ** *p* < 0.01, *** *p* < 0.001, and **** *p* < 0.0001 versus the vehicle group (one-way ANOVA plus Tukey’s multiple comparisons test).

To investigate the antiviral effects of the compounds on HCoV-OC43-infected cells, the expression levels of IFN-related antiviral genes (IFN-α1, IFN-β1, and IFN-λ1) and MxA were evaluated. HCoV-OC43 infection induced the expression of IFN-related antiviral genes in MRC-5 cell lysates at 1, 2, 3, and 4 dpi (Figure 6A–C). Compound treatment following virus infection reduced the mRNA expression levels of IFN-α1, IFN-β1, and IFN-λ1. In addition, MxA mRNA expression was increased by compound treatment at one dpi as compared with virus-infected MRC-5 cells without compound treatment. However, at a later stage of infection, MxA mRNA levels following compound treatment were lower than those of the vehicle control (Figure 6D).

**Figure 7:**
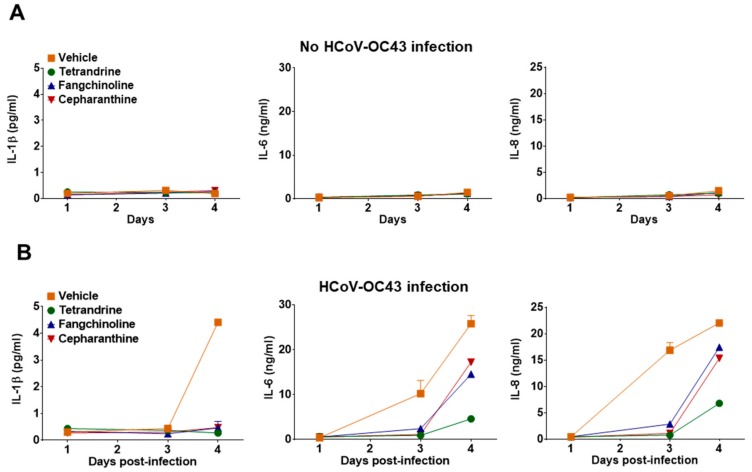
Reduced inflammatory cytokine production by treatment with TET, FAN, and CEP in HCoV-OC43-infected MRC-5 cells. (**A** and **B**) MRC-5 cells with or without HCoV-OC43 infection were treated with vehicle (0.025% DMSO), or the compounds (5 μM). The secretion of inflammatory cytokines IL-1β, IL-6, and IL-8 was quantified by CBA in the compound-treated virus-uninfected supernatant (**A**) and the compound-treated virus-infected supernatant (**B**) at 1, 3, and 4 dpi. Data are presented as means ± SEM of three independent experiments.

To determine whether TET, FAN, and CEP modulate cytokine expression in HCoV-OC43-infected cells, the kinetics of various inflammatory cytokines (i.e., TNF, IL-1β, IL-6, IL-8, IL-10, and IL-12p70) in the cell culture supernatant were evaluated at four dpi. Notably, TNF, IL-10, and IL-12p70 were not detected in the MRC-5 cell culture supernatant following compound treatment (data not shown). Additionally, treatment with the compounds did not induce MRC-5 to produce the inflammatory cytokines IL-1β, IL-6, and IL-8 (Figure 7A). As shown in Figure 7B, the expression levels of IL-1β, IL-6, and IL-8 were significantly upregulated by virus infection, but were reduced in the compound-treated group. These data indicate that HCoV-OC43 induced an antiviral response, and inflammatory cytokine production was reduced by treatment with TET, FAN, and CEP.

### 3.5. TET Promoted the Phosphorylation of P38 Mitogen-Activated Protein Kinase (MAPK) in HCoV-OC43-Infected MRC-5 Cells, but Enhanced P38 Phosphorylation by ANM Had no Antiviral Synergistic Effect with TET

**Figure 8:**
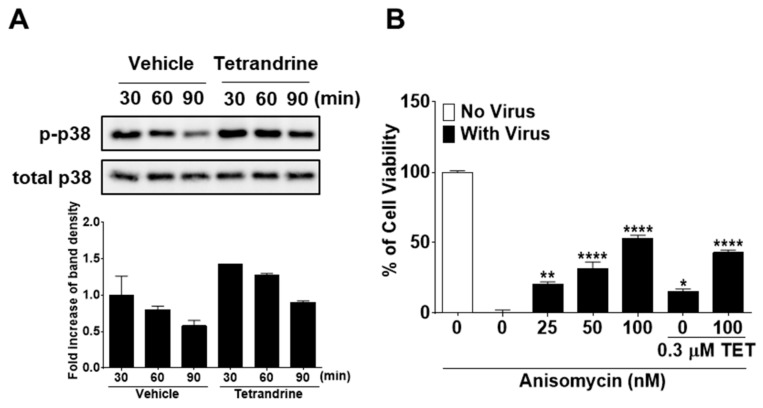
Increased phosphorylation of p38 mitogen-activated protein kinase (MAPK) by TET treatment in HCoV-infected MRC-5 cells with no antiviral synergistic effect of TET and anisomycin. (**A**) Cells were infected with HCoV-OC43, and 5 μM TET (vehicle 0.025% DMSO) was added to the cell cultures. Cell lysates were harvested at 30, 60, and 90 min post-infection. Western blot analysis was performed to determine the extent of p38 phosphorylation (Thr180/Tyr182). Total p38 was used as a control. (**B**) MRC-5 cells treated with 25, 50, or 100 nM ANM were infected with the virus in the presence or absence of 0.3 μM TET. Cell viability was assessed using the MTS assay at four dpi. Data are presented as mean ± SEM of three independent experiments. * *p* < 0.05, ** *p* < 0.01, *** *p* < 0.001, and **** *p* < 0.0001 versus the vehicle group (one-way ANOVA plus Tukey’s multiple comparisons test).

Several studies have reported that TET enhances the phosphorylation of p38 MAPK [25,26]. Therefore, to further elucidate the mode of action of TET in response to HCoV-OC43 infection, we investigated whether the p38 MAPK signaling pathway is activated by TET in virus-infected MRC-5 cells. The results showed that in HCoV-OC43-infected MRC-5 cells, p38 phosphorylation peaked at 30 min post-infection and then slowly decreased at 60 and 90 min. TET treatment induced an increase in the phosphorylation of p38 MAPK as compared with the vehicle-treated group (Figure 8A). To determine whether the activation of p38 MAPK has a role in the antiviral responses of host cells, virus-infected MRC-5 cells were treated with the MAPK signal activator ANM with and without 0.3 μM TET. As shown in Figure 8B, virus-induced cell death was decreased by ANM treatment in a dose-dependent manner with no indication of cytotoxicity, although the addition of ANM did not fully recover cell viability even at a concentration of 100 nM. However, the viability of HCoV-OC43-infected MRC-5 cells was similar to 100 nM ANM + 0.3 μM TET versus 100 nM ANM, suggesting that there was no synergistic effect of TET and ANM against HCoV-OC43. Collectively, these data indicate that TET induced the activation of p38 MAPK in HCoV-OC43-infected MRC-5 cells, and although the MAPK signal activator ANM had a partial antiviral effect, there was no synergistic effect of TET and ANM.

## 4. Discussion

HCoVs, including strain OC43, are known to cause 15–30% of mild upper respiratory tract infections in humans [27]. Considering the recent outbreak of new highly pathogenic HCoVs, such as SARS-CoV and MERS-CoV, further understanding the HCoV life cycle and the development of effective treatments are urgently needed. However, the manipulation of SARS-CoV and MERS-CoV for diagnostic, culture, and research purposes should be carried out under BSL-3 conditions [28,29] indicating a limitation to research accessibility. Several recent studies have shown that the genome of HCoV-OC43 encodes some well-conserved motifs shared with SARS-CoV, suggesting that HCoV-OC43 could be an alternative model for the study of emerging HCoVs, which should be conducted in a BSL-3 facility [22].

The results of the present study demonstrated that TET, FAN, and CEP, which have been widely applied in antitumor studies, are potentially strong natural antiviral agents for the treatment of HCoV infection. These compounds had no cytotoxic effects on MRC-5 cells at concentrations of up to 10 μM (CC_50_ > 10 μM) (Figure 1), and co-treatment considerably inhibited HCoV-OC43-induced cell death in a dose-dependent manner (Figure 2). The approximate IC_50_ values of TET, FAN, and CEP were 0.33 ± 0.03, 1.01 ± 0.07, and 0.83 ± 0.07 μM, respectively, with selective indices of > 40.19, 11.46, and 13.63. Previous studies reported antiviral IC_50_ values of 55 nM for TET against Ebola virus [9], 0.8–1.7 μM for FAN against HIV-1 [10] and 26.4 nM for CEP against HIV-1 [30], indicating that the effective doses of these compounds vary.

To understand the modes of action of TET, FAN, and CEP against HCoV-OC43 infection, concentrations of 0.2, 1, and 5 μM were added to MRC-5 cell cultures at 24 h before (pre-treatment), during (co-treatment), and after (post-treatment) virus infection. These compounds protected against virus-induced cell death in a dose-dependent manner. In the pre- and co-treatment groups, cell survival was considerably increased as compared with the post-treatment group, suggesting differences in the time-dependent efficiencies of TET, FAN, and CEP (Figure 3). To further understand the time course of the TET-, FAN-, and CEP-mediated antiviral effects, 5 μM concentrations of the compounds were added at different time points to the cultures of HCoV-OC43-infected MRC-5 cells. Pretreatment with the compounds highly increased the cell survival rates against virus infection. Of particular interest, only 3 h of pre-exposure to TET and FAN fully protected against virus-induced cell death. In contrast, delayed compound treatment did not increase the viability of virus-infected MRC-5 cells as compared with the pre- and co-treatment groups (Figure 4), strongly demonstrating that TET, FAN, and CEP are more effective at the early stage of virus infection.

To further verify the antiviral effect of TET, FAN, and CEP, the kinetics of HCoV-OC43 replication in the supernatants and cell lysates of infected MRC-5 cells were examined. As shown in Figure 5A–C, the virus copy number was dramatically increased in the cell lysates at 1–2 dpi and in the supernatants at 2–3 dpi, indicating that the virus particles might start budding into the supernatant from two dpi, at which time the compounds no longer had antiviral effects. The virus particles were considerably inhibited by TET, FAN, and CEP, not only in the supernatants but also in the cell lysates. In addition, the expression levels of the S and N proteins in MRC-5 cells at two dpi were significantly diminished by TET, FAN, and CEP treatment (Figure 5D,E).

The S protein plays an important role in viral entry and as a determinant of CoV tropism [31]. The S protein of HCoV-OC43 recognizes 9-O-acetylated sialic acid as a receptor determinant [32,33], and HCoV-OC43 expresses hemagglutinin esterase, which has sialic acid-9-O-acetyltransferase activities as a receptor-destroying enzyme [34]. In this study, HCoV-OC43 infection induced a significant increase in the mRNA level of SIAE in MRC-5 cells at four dpi as a virus-induced host response. However, SIAE mRNA levels were lowered by treatment with TET, FAN, and CEP (Figure 5F). Collectively, these data suggest that TET, FAN, and CEP inhibited virus replication, viral protein expression, and the virus-induced host response in HCoV-OC43-infected MRC-5 cells.

To determine the effects of these compounds on the host antiviral response, IFN-related antiviral genes and inflammatory cytokine production were measured in HCoV-OC43-infected MRC-5 cells [35,36,37]. As shown in Figure 6A–D, the mRNA levels of IFN-α1, IFN-β1, IFN-λ1, and MxA were significantly increased in the lysates of virus-infected cells. However, the expression levels of the IFN-related antiviral genes were remarkably lower in compound-treated virus-infected MRC-5 cells as compared with the virus-infected group without compound treatment. Especially, MxA mRNA levels were significantly increased by compound treatment as early as one dpi, although the mRNA expression levels of MxA were significantly decreased by compound treatment at four dpi. Hence, these data suggest that these compounds induced MxA mRNA expression at the early stage of HCoV-OC43 infection. Among the several investigated inflammatory cytokines, the IL-1β, IL-6, and IL-8 levels were increased in MRC-5 cells by HCoV-OC43 infection, but were relatively low in compound-treated virus-infected MRC-5 cells as compared with the virus-infected group without compound treatment (Figure 7). In addition, the production of inflammatory cytokines was not affected by the compound treatment dose.

A recent study reported that TET activates the MAPK signaling pathway [26]. Likewise, the results of the present study showed that TET treatment promotes the phosphorylation of p38 MAPK in HCoV-OC43-infected MRC-5 cells (Figure 8). To investigate the influence of the activation of the MAPK signaling pathway by TET treatment on the antiviral effects of TET, ANM was added to the culture of virus-infected cells in the presence and absence of TET. The results showed that virus-induced cell death was reduced by up to 52% following the addition of ANM, suggesting a dose-dependent effect of ANM. However, the addition of 100 nM ANM plus 0.3 μM TET did not increase the survival of MRC-5 cells as compared with that of 100 nM ANM treatment only. Collectively, these data suggest that activation of the MAPK signaling pathway decreased virus-induced cell death, but there was no synergistic antiviral effect with the combination of TET and ANM.

In summary, the results of the present study demonstrate that TET, FAN, and CEP inhibited the infectivity of HCoV-OC43 in MRC-5 cells at the early stage of infection. Furthermore, TET, FAN, and CEP inhibited the replication of HCoV-OC43, viral protein expression, and the virus-induced host response of MRC-5 cells. These findings establish TET, FAN, and CEP as natural compounds that may be useful for the prevention and treatment of HCoV infections.

## 5. Conclusions

This study investigated the antiviral effects of the natural substances TET, FAN, and CEP against HCoV-OC43 infection of MRC-5 cells. TET, FAN, and CEP significantly inhibited virus-induced cell death with no apparent cytotoxicity at the effective doses. Pretreatment with these compounds efficiently increased the viability of MRC-5 cells to the same extent as co-treatment. These results indicate that TET, FAN, and CEP can be applied for the prevention and treatment of HCoV infection. Treatment with TET, FAN, and CEP dramatically suppressed the replication of HCoV-OC43 in the supernatants and lysates of MRC-5 cells, the expression of viral S and N proteins, and the virus-induced host response. Moreover, TET induced the activation of the p38 MAPK pathway in HCoV-OC43-infected MRC-5 cells, and although the p38 activator ANM conveyed a partial antiviral effect, there was no synergistic effect with the addition of ANM. Taken together, these results demonstrate the potential of TET, FAM, and CEP for the prevention and treatment of HCoV-OC43 infection.

## Figures and Tables

**Table 1:** Sequences of the human-specific primers used for qRT-PCR.

Gene (Human)	Sense (5′→3′)	Antisense (5′→3′)	Product Size (bp)
MxA	CAACCTGTGCAGCCAGTATG	GTCCTGCTCCACACCTAGAG	85
IFN-α1	GTGCTCAGCTGCAAGTCAAG	TTATCCAGGCTGTGGGTCTC	65
IFN-β1	ACCAACAAGTGTCTCCTCCA	GTAGTGGAGAAGCACAACAGG	50
IFN-λ1	GTCACCTTCAACCTCTTCCG	TCAGACACAGGTTCCCATCG	70
SIAE	TATGAACACCGTCTCCACCC	CAACCACAGTGCCATGACAA	65
β-Actin	GGAAATCGTGCGTGACATCA	ATCTCCTGCTCGAAGTCCAG	63

## Abbreviations

CEP	Cepharanthine
Dpi	Days post-infection
FAN	Fangchinoline
HCoV-OC43	Human coronavirus OC43
IFN	Interferon
IL	Interleukin
TET	Tetrandrine
TNF	Tumor necrosis factor
